# A stratified flow of a non-Newtonian Casson fluid comprising microorganisms on a stretching sheet with activation energy

**DOI:** 10.1038/s41598-023-38260-0

**Published:** 2023-07-11

**Authors:** Showkat Ahmad Lone, Sadia Anwar, Anwar Saeed, Gabriella Bognár

**Affiliations:** 1 Department of Basic Sciences, College of Science and Theoretical Studies, Saudi Electronic University, 11673 Riyadh, Jeddah-M Kingdom of Saudi Arabia; 2 Department of Mathematics, College of Arts and Sciences, Prince Sattam Bin Abdul Aziz University, 11991 Wadi ad Dawasir, Al-Kharj Kingdom of Saudi Arabia; 3 Center of Excellence in Theoretical and Computational Science (TaCS-CoE), Science Laboratory Building, Faculty of Science, King Mongkut’s University of Technology Thonburi (KMUTT), 126 Pracha-Uthit Road, Bang Mod, Thung Khru, Bangkok, 10140 Thailand; 4 Institute of Machine and Product Design, University of Miskolc, Miskolc-Egyetemvaros, 3515 Hungary

**Keywords:** Engineering, Mathematics and computing

## Abstract

A stratified flow may be seen regularly in a number of significant industrial operations. For instance, the stratified flow regime is typically used by gas-condensate pipelines. Clearly, only a limited set of working situations for which this flow arrangement is stable allow for the achievement of the stratified two-phase flow zone. In this paper, the authors are considered the laminar, steady and incompressible magnetohydrodynamic flow of a non-Newtonian Casson fluid flow past a stratified extending sheet. The features of bio-convection, Brownian motion, thermal radiation thermophoresis, heat source, and chemically reactive activation energy have been employed. The set of equations administered flow of fluid is converted into ordinary differential equation by suitable variables. A semi-analytical investigation of the present analysis is performed with homotopy analysis method. Endorsement of the current results with previous results is also investigated. The outcomes showed that the velocity distribution of the fluid flow lessens with higher Casson and magnetic factors. The temperature profiles of fluid flow shrinkage as the Prandtl number and Casson factor increase and enlarges with higher values of thermal radiation, magnetic, and Brownian motion factors. It is found that the growing thermophoretic and Brownian motion factors reduce the rate of thermal flow of the Casson fluid flow. In contrast, the increasing thermal stratification parameter increases the thermal flow rate of fluid.

## Introduction

Fluids that disobey the Newtonian law of viscosity are termed as non-Newtonian fluids such as ketchup, honey, pasts, paints, gels, and polymer solution etc. Many of their applications include printing technologies, food products, dragging reducing agent, and polymer fluid flow through pipes at industrial level etc. Shah et al.^1^ have explored gold-blood nanofluid flow amid two porous plates by considering micropolar effects upon the flow system and determined that fluid linear motion has opposed by microrotation factor whereas the rotational motion has augmented in this process. Salahuddin et al.^2^ analyzed permeable squeezing flow of Maxwell fluid with thermally radiative and chemically reactive effects and have proved that velocity distribution has weakened while thermal distribution has boosted for hike in porosity factor. Sarada et al.^3^ revealed the effects of MHD on non-Newtonian fluid past an extending sheet and have established that velocity panel has deteriorated and temperature has amplified for progression in magnetic factor. Shehzad et al.^4^ studied MHD non-Newtonian fluid flow past an inclined permeable as well as rotating plate. Abiev^5^ has studied mathematically the bi-phase hydrodynamics Taylor flow for different fluids through a conduit and has matched his results with published works with a fine agreement amongst all results. Banerjee et al.^6^ have inspected the effects of electro-viscous flow of non-Newtonian fluid in a channel with slip condition at higher zeta potential. Gautam et al.^7^ considered MHD bio-convective non-Newtonian fluid flow subject to the impact of multiple slip conditions and nonlinear thermal radiations. Kumar and Sahu^8^ have discussed the non-Newtonian flow of fluid on a spinning cylinder in a regime of flow of fluid and deliberated that the lift and drag coefficients have declined with augmentation in Reynolds number and rotation speed. He et al.^9^ have explored the dynamics of mixed convective and thermally radiative non-Newtonian fluid flow on a surface using power law velocity slip condition along with Hall current and proved that thermal characteristics enlarged with progression in radiation factor. Archana et al.^10^ inspected Casson squeezing nanofluid flow subject to time variations, slip conditions, and magnetic effects and have establish that the velocity panel has heightened for greater squeezing factor, while the thermal panel exhibited an identical performance for thermophoresis and Brownian motion factors. Ganesh^11^ scrutinized nonlinearly radiative flow of nanofluid in 3D space on an exponentially elongating surface and has solved the modeled problem computationally. Kumar et al.^12^ considered cross diffusive properties for mixed convective MHD fluid flow with impacts of nonlinear radiation on a vertical surface and have noted that growth in Soret as well as Dufour factors have upsurge the concentration and thermal distributions. Kumar et al.^13^ evaluated the impressions of convective constraints and uniform heat sink/source on nanofluid flow using Marangoni convective effects and have noted that with larger values of heat source, maximum heat is added to the system that has augmented the thermal distribution. Zeeshan et al.^14^ conducted a thermal analysis for nanofluid flow (of non-Newtonian nature) on a parabolic curve using chemical reaction and deduced that thermal distribution has weakened with upsurge in Casson factor while it has boosted with advancement in chemically reactive factor. Salahuddin et al.^15^ examined the variations in thermophoresis properties for Carreau fluid flow on a parabolic elongating surface with impacts of heat generation and proved that thermal panels have amplified with progression in heat generation, while upsurge in Prandtl number has an adverse impact on heat transmission. Salahuddin et al.^16^ evaluated the impacts of Dufour/Soret impacts on penetrable flow of Carreau fluid on a thermally radiative cylinder. Waqas^17^ analyzed chemically reactive effects and dual diffusions for a liquid (non-Newtonian) on a stretching surface and has ensured the convergence and validation of his problem solution by matching his results with already established data. Salahuddin et al.^18^ inspected the transportation phenomenon for 2D cross nanofluid flow on a parabolic surface using the mass and heat flux model proposed by Cattneo-Christov and highlighted that thermal and concentration distributions have weakened for progression in corresponding relaxation factors.

The collective impact of free and forced convection is generally termed as mixed convection. It plays a substantial part in numerous engineering uses for instance solar collectors, electronic equipment and nuclear reactors. Such a process occurs whenever the influence of buoyancy force is more substantial in the forced convective process or the influence of forced flow in the free convective process becomes more dominant. Wahid et al.^19^ discussed computationally the mixed convective fluid flow at three dimensional stagnant point of vertical plate and have determined that velocity of fluid has weakened while temperature has enlarged with expansion in nanoparticles concentration. Qureshi et al.^20^ computationally simulated MHD mixed convective fluid flow in a conduit with cavities and exposed that by improving the radius of channel the thermal flow in the channel has enhanced by 119%. Islam et al.^21^ explored mixed convective nanofluid flow on an elongating cylinder with the impact of thermal source as well as sink and have established that concentration has deteriorated while temperature has risen with progress in Brownian motion factor. Al-Hassani et al.^22^ have simulated mixed convective nanofluid flow in a triangular cavity by keeping the bottom of cavity as insulated while the inclined wall has kept at some fixed temperature. Patel^23^ has explored the thermal production influences upon mixed convective MHD fluid flow at the stagnation point of permeable medium. Fu et al.^24^ have analyzed comprehensively the mixed convective nanofluid flow over a surface and have discussed the influences of various emerging factors on flow distributions. The readers can further have an insight of related concept in Refs.^25–30^.

Fluids that are conducted electrically such as salted water and plasma etc. are named as magnetohydrodynamic (MHD). Many of their applications are comprised of the areas of biomedical engineering, medical sciences, chemical engineering, and fluid dynamics etc. The main benefit of applying the principles of MHD is to divert the flow filed in the desired direction by shifting the boundary layer development. The theory of MHD was first introduced by Hartmann^31^. Waqas et al.^32^ considered the thermally radiative MHD fluid flow on a stratified convective sheet and has deduced that the thermal distribution and Nusselt number have amplified with boosted values of curvature factor. Jamshed et al.^33^ physically specified the MHD mixed convection nanofluid flow through the inner elliptic cylinder and have deduced that Hartmann number has a positive impact upon thermal characteristics. Asjad et al.^34^ evaluated the impacts of activated energy over MHD fluid flow past a elongating surface using the impact of microorganism and have explored that velocity of fluid has boosted with upsurge in mixed convection and magnetic parameters. Bejawada et al.^35^ have examined the influences of radiations upon MHD fluid flow past a surface using Forchheimer permeable surface. Kodi and Mopuri^36^ examined the MHD fluid flow on a permeable surface and proved that velocity distribution has decayed with expansion in inclination angle, magnetic, and Casson factors. Sharma et al.^37^ explored the convection MHD fluid flow on an extended rotary disk with Soret and Dufour effects. Guedri et al.^38^ discussed EMHD fluid flow past a widening sheet and realized that thermal panels have enlarged with for growth in magnetic and electric factors. Waqas et al.^39^ simulated a modified model for nanofluid flow at stagnation point of cross fluid on an elongating and shrinking cylinder and deduced that heat and mass diffusions have affected by curvature of cylinder as well as heat source and sink factor. Waqas et al.^40^ inspected the dual stratification and chemically reactive effects on MHD Jeffery fluid flow subject to the impacts of heat sink-source, and thermally radiative effects.

Thermophoresis and Brownian motion phenomena are the mechanisms of mass as well as thermal transmission of tiny particles in a manner of reducing the concentration and temperature gradients that also influenced these tiny particles associated with bulk surfaces. These are the two substantial sources for migration of fluid particles. Thermophoresis and Brownian motion have many applications in different fields such as nuclear safety phenomena, hydrodynamics, atmospheric pollution, and aerosol technology etc. Pasha et al.^41^ have applied the analytical approaches for discussing the influences of magnetic factor and Brownian motion as well as thermophoresis effects amid two plates and explored that thermal flow has been upsurge for progression in Brownian and thermophoretic factors. Saghir and Rahman^42^ have explored Brownian motion and thermophoresis effects over fluid flow in a channel and have deduced that diameter of nanoparticles has more impact upon the thermal diffusion enhancement. Soomro et al.^43^ have discussed computationally the impression of Brownian motion and thermophoresis by using Crank-Nicolson approach for solution of modeled equations. Shah et al.^44^ have discussed diffusions effects of thermophoresis and Brownian motion on upper convective Maxwell nanofluid flow over vertical shaped surface and have confirmed that enhancement in Brownian factor has promoted thermal conductance and motion of nanoparticles. Harish and Sivakumar^45^ have exposed the influence of nanoparticles distribution on fluid flow through an enclosure taking the effects of thermophoresis and Brownian motion in the fluid flow system. Kalpana et al.^46^ have studied the MHD hybrid nanofluid flow in irregular shaped channel using the influences of Brownian motion and thermophoresis and have explored that fluid’s thermal profiles have been amplified with upsurge in magnetic factor, volume faction of nanoparticles and Brownian motion factor. Hazarika and Ahmad^47^ have explored the behavior of thermophoresis and Brownian motion on nanoparticles flow and have explored that the growing diameter of nanoparticles has enhanced the Brownian motion within the flow system.

Microorganisms like microalgae and bacteria are comparatively denser than water and subsequently capable to swim in reverse direction of gravity. During this phenomenon high magnitude of microorganisms are accumulating at the upper surface of suspension and are causing a disturbance in density of upper and lower layers of suspension. As a result a convection pattern is initiated due to the convective instability in aforementioned phenomenon. Such random motion is responsible for occurrence of bioconvection in the fluid flow process and has many practical applications such as ecological products like ethanol, fuels and fertilizers etc. Eldabe et al.^48^ studied nanofluid flow using gyrotactic microorganisms and thermophoresis as well as Brownian motion and revealed that thermal flow panels have amplified with impact of magnetic factor and Brownian motion parameter. Ijaz et al.^49^ have simulated fluid flow in a vacuum using magnetic field, nonlinear thermal radiations and gyrotactic microorganisms. Bhatti et al.^50^ have investigated MHD Williamson nanoparticles flow amid rotary circular plates induced in a permeable medium subject to the influences of gyrotactic microorganisms. Alrabaiah et al.^51^ have assessed parametrically the microorganism fluid flow amid conical gap of rotary disk and cone. Madhukesh et al.^52^ have explored the dynamics of swimming microorganism and water-based nanofluid flow on a Riga plate with effect of thermal source and sink and have estimated that upsurge in slip effects has declined the profiles of concentration, temperature and velocity of fluid. Azam^53^ has exposed numerically the mathematical model of bioconvective time-based nanofluid flow on a surface with nonlinear radiations and explored that fluid motion has deteriorated for expansion in bioconvective Rayleigh number. Azam et al.^54^ designed mathematically a new model to investigate the impact of bio-convection and activation energy on chemically reactive nanofluid flow using nonlinearly radiative effects and have deduced that the microorganism motile number has dropped for progression in Peclet number and variance factor of microorganism. Waqas et al.^55^ studied bio-convective MHD stratified nanofluid flow supported by gyrating and elongating sheet using dissipative and Joule heating effects.

Keeping in mind the above literature, we are sure that there is very less work based on the stratified flow of a non-Newtonian Casson fluid flow over a stretching surface. For liquid–gas and liquid–liquid two-phase flow in a gravitational environment, stratified flow is a fundamental flow configuration in which the lightened fluid flows over the thicker one. This flow pattern may be seen regularly in a number of significant industrial operations. There are two-phase phenomena known as stratified and slug flows, which occur in many applications, such as petroleum transportation and chemical microreactors. Along a microchannel, the slug flow reduces the transfer distance and enhances the mixing process. The pressure drop in production pipelines is heavily influenced by phase flow rates, pipe diameters, and fluid properties such as density, viscosity and surface tension. Therefore, the flow is considered to be incompressible, laminar, and steady. Various flow conditions have been employed for current problem. The analysis is considered in the following subsequent sections. In “Problem formulation” section, the model formulation is presented. A semi-analytical investigation along with validation with previous results is presented in “HAM solution” section. “Validation” section describes the discussion of various results of the present analysis whereas “Discussion of results” section includes the outcomes of this study.

## Problem formulation

**Figure 1:**
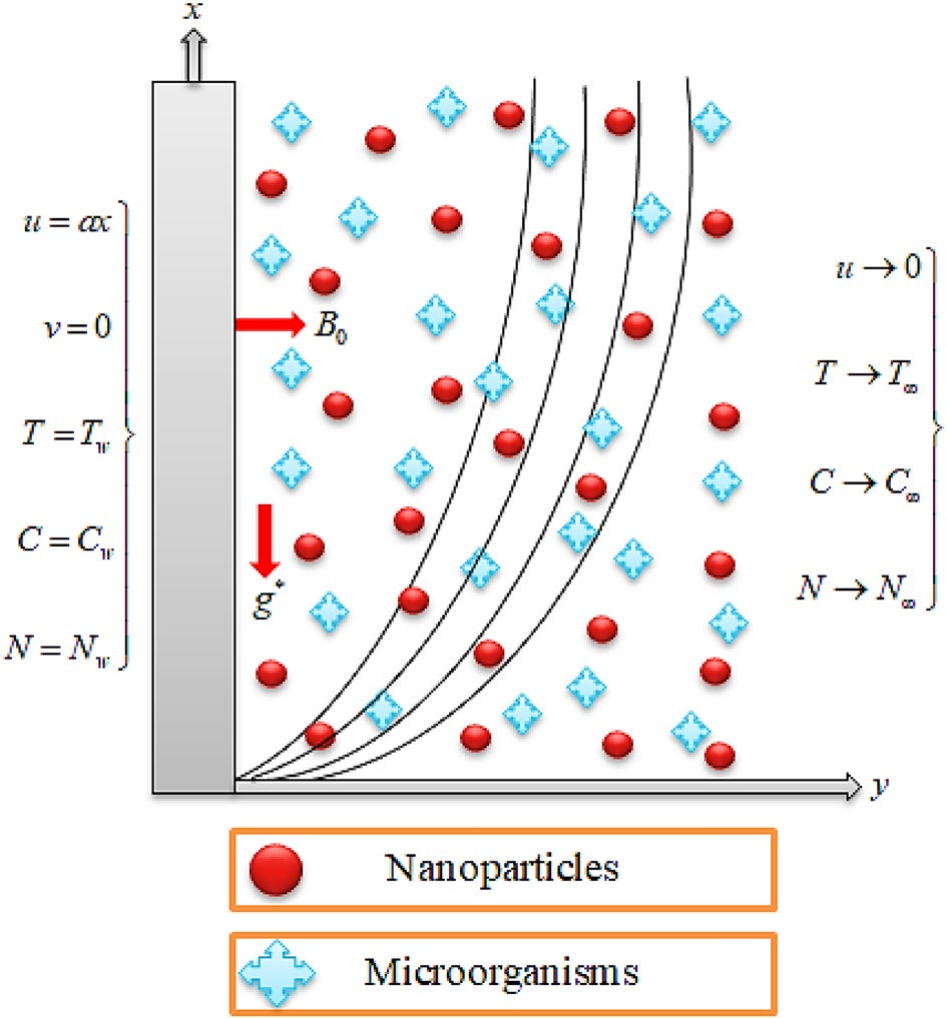
Geometrical view of flow scheme.

Assume the two-dimensional magnetohydrodynamic flow of a non-Newtonian Casson fluid on a stratified stretching sheet. The features of bioconvection and thermophoresis phenomena have been used along with effects of heat source, thermal radiation, activation energy and chemical reaction. The stretching velocity along *x*-axis is denoted by $u_{w} = a\,x$ with $a > 0$ as constant and *y*-axis as in normal direction. $B_{0}$ is the strength of magnetic effects that is taken normal to flow direction. Temperature at surface and its ambient values are $T_{w}$ and $T_{\infty }$. Likewise the surface nanoparticle and microorganisms concentration are dented by $C_{w}$ and $N_{w}$, respectively. The ambient nanoparticle and microorganisms concentration are dented by $C_{\infty }$ and $N_{\infty }$. The geometrical representation of two-dimensional Cartesian coordinate system is described in Fig. 1. Using the suppositions, the flow equations take the subsequent form:

1 $$T_{ij} = \left\{ {\begin{array}{*{20}l} {\left( {2\mu_{{{\text{Bnf}}}} + \frac{{p_{y} }}{{\sqrt {\left( {\pi_{c} /2} \right)} }}} \right)e_{mn} ,\,} \hfill & {\pi_{c} > \pi ,} \hfill \\ {\left. {2\mu_{{{\text{Bnf}}}} + \frac{{p_{y} }}{{\sqrt {\left( {\pi_{c} /2} \right)} }}} \right)e_{mn} ,} \hfill & {\pi_{c} < \pi ,} \hfill \\ \end{array} } \right.$$

Above $\pi_{c}$ shows the critical value of $\pi$, $e_{mn}$ is the $\left( {m,n} \right)th$ deformation rate, $\mu_{{{\text{Bnf}}}}$ represents dynamic viscosity of plastic while yield stress is given by $P_{y}$.

The mass conservation, momentum, energy, nanoparticle concentration and microorganisms concentration equations can be described as:

2 $$\nabla \cdot {\mathbf{V}} = 0,$$

3 $$\rho_{f} \left( {{\mathbf{V}} \cdot \nabla {\mathbf{V}}} \right) = - \nabla p + \mu \left( {1 + \frac{1}{\beta }} \right)\nabla^{2} {\mathbf{V}} - \frac{{\sigma_{f} B_{0}^{2} }}{{\rho_{f} }}u + g^{*} \left[ {\begin{array}{*{20}l} {\beta^{*} \left( {T - T_{\infty } } \right) - \frac{{M_{p} \left( {\rho_{p} - \rho_{f} } \right)}}{{\rho_{p} \rho_{f} }}} \hfill \\ { \times \left( {C - C_{\infty } } \right) - \gamma^{*} \frac{{\left( {\rho_{m} - \rho_{f} } \right)}}{{\rho_{f} }}\left( {N - N_{\infty } } \right)} \hfill \\ \end{array} } \right],$$

4 $$\left( {\rho C_{p} } \right)_{f} \left( {{\mathbf{V}} \cdot \nabla T} \right) = k\nabla^{2} T - \nabla q_{r} + \frac{{Q_{0} }}{{\left( {\rho C_{p} } \right)_{f} }}\left( {T - T_{\infty } } \right) + \left( {\rho C_{p} } \right)_{p} \left( {\frac{{M_{p} D_{B} }}{{\rho_{p} }}D_{B} \nabla T.\nabla C + \left( {\frac{{D_{T} }}{{T_{\infty } }}} \right)\,\,\nabla T.\,\nabla T} \right),$$

5 $$\left( {\rho C_{p} } \right)_{f} \left( {{\mathbf{V}} \cdot \nabla C} \right) = D_{B} \nabla^{2} C + \frac{{\rho_{p} D_{T} }}{{M_{p} T_{\infty } }}\nabla^{2} T - K_{r} \left( {C - C_{\infty } } \right)\left( {\frac{T}{{T_{\infty } }}} \right)^{n} \exp \left( { - \frac{{E_{a} }}{{k_{B} T}}} \right),$$

6 $${\mathbf{V}} \cdot \nabla N = D_{m} \nabla^{2} N - \frac{{bW_{C} }}{{\left( {C_{w} - C_{\infty } } \right)}}\nabla \left( {N \cdot \nabla C} \right),$$

Hear the velocity vector is ${\mathbf{V = }}\left( {u,v} \right)$ and $\beta$ is the Casson factor. From above we have:

7 $$\frac{\partial \,u}{{\partial \,x}}\,\, + \,\,\,\frac{\partial \,v}{{\partial \,y}} = 0,\,$$

8 $$u\frac{\partial u}{{\partial x}} + v\frac{\partial u}{{\partial y}} = \nu_{f} \left( {1 + \frac{1}{\beta }} \right)\frac{{\partial^{2} u}}{{\partial y^{2} }} - \frac{{\sigma_{f} B_{0}^{2} }}{{\rho_{f} }}u + g^{*} \left[ {\beta^{*} \left( {T - T_{\infty } } \right) - \gamma^{*} \frac{{\left( {\rho_{m} - \rho_{f} } \right)}}{{\rho_{f} }}\left( {N - N_{\infty } } \right) - \frac{{M_{p} \left( {\rho_{p} - \rho_{f} } \right)}}{{\rho_{p} \rho_{f} }}\left( {C - C_{\infty } } \right)} \right],$$

9 $$u\frac{\partial T}{{\partial x}} + v\frac{\partial T}{{\partial y}} = \left( {\frac{{k_{f} }}{{\left( {\rho C_{p} } \right)_{f} }} + \frac{1}{{\left( {\rho C_{p} } \right)_{f} }}\frac{{16\sigma^{*} T_{\infty }^{3} }}{{3k^{*} k_{f} }}} \right)\frac{{\partial^{2} T}}{{\partial y^{2} }} + \frac{{Q_{0} }}{{\left( {\rho C_{p} } \right)_{f} }}\left( {T - T_{\infty } } \right) + \frac{{\left( {\rho C_{p} } \right)_{p} }}{{\left( {\rho C_{p} } \right)_{f} }}\left( {\frac{{M_{p} D_{B} }}{{\rho_{p} }}\frac{\partial C}{{\partial y}}\frac{\partial T}{{\partial y}} + \frac{{D_{T} }}{{T_{\infty } }}\frac{{\partial^{2} T}}{{\partial y^{2} }}} \right),$$

10 $$u\frac{\partial C}{{\partial x}} + v\frac{\partial C}{{\partial y}} = D_{B} \frac{{\partial^{2} C}}{{\partial y^{2} }} + \frac{{\rho_{p} D_{T} }}{{M_{p} T_{\infty } }}\frac{{\partial^{2} T}}{{\partial y^{2} }} - K_{r}^{2} \left( {C - C_{\infty } } \right)\left( {\frac{T}{{T_{\infty } }}} \right)^{n} \exp \left( { - \frac{{E_{a} }}{{k_{B} T}}} \right),$$

11 $$u\frac{\partial N}{{\partial x}} + v\frac{\partial N}{{\partial y}} = D_{m} \frac{{\partial^{2} N}}{{\partial y^{2} }} - \frac{{bW_{C} }}{{\left( {C_{w} - C_{\infty } } \right)}}\left( {N\frac{{\partial^{2} C}}{{\partial y^{2} }} + \frac{\partial C}{{\partial y}}\frac{\partial N}{{\partial y}}} \right),$$

The conditions at the boundaries are:

12 $$\left\{ {\begin{array}{*{20}l} {u = u_{w} \left( x \right),\,\,\,v = 0,\,\,\,\,\,T = T_{w} ,\,\,C = C_{w} ,\,\,N = N_{w} \,\,\,at\,\,y = 0,} \hfill \\ {u \to 0,\,\,\,\,\,\,\,C \to C_{\infty } ,\,\,\,\,T \to T_{\infty } ,\,\,\,N \to N_{\infty } \,\,\,as\,\,\,y \to \infty .} \hfill \\ \end{array} } \right\}$$

The stratified restrictions are defined as:

13 $$\left\{ \begin{gathered} T_{w} = T_{0} + e_{1} x,\,\,\,\,T_{\infty } = T_{0} + d_{1} x \hfill \\ C_{w} = C_{0} + e_{2} x,\,\,\,\,C_{\infty } = C_{0} + d_{2} x \hfill \\ N_{w} = N_{0} + e_{3} x,\,\,\,\,N_{\infty } = N_{0} + d_{3} x \hfill \\ \end{gathered} \right\},$$

where $e_{1}$, $e_{2}$, $e_{3}$, $d_{1}$, $d_{2}$ and $d_{3}$ are positive constants.

The variables of transformation are given by^56,57^:

14 $$\left\{ {\begin{array}{*{20}l} {u = axf^{\prime}\left( \eta \right),\,\,\,\,v = - \sqrt {a\nu_{f} } f\left( \eta \right),\,\,\,\,T = T_{\infty } + \left( {T_{w} - T_{\infty } } \right)\,\,\theta \left( \eta \right),} \hfill \\ {N = N_{\infty } + \left( {N_{w} - N_{\infty } } \right)\,\,\,\chi \left( \eta \right),\,\,\,\,C = C_{\infty } + \,\,\left( {C_{w} - C_{\infty } } \right)\,\,\,\phi \left( \eta \right),\,\,\,\,\eta = y\sqrt {\frac{a}{{\nu_{f} }}} .} \hfill \\ \end{array} } \right\}$$

By incorporating Eq. (14) we have from above:

15 $$\left( {1 + \frac{1}{\beta }} \right)f^{\prime\prime\prime} + f\,f^{\prime\prime} - Mf^{\prime} + \omega \left( {\theta - Rb\phi - Rc\chi } \right) - \left( {f^{\prime}} \right)^{2} = 0,$$

16 $$\left( {1 + Rd} \right)\,\,\theta^{\prime\prime}f + \Pr \left( {Nb\phi^{\prime}\theta^{\prime} + \theta^{\prime} + Nt\left( {\theta^{\prime}} \right)^{2} - S_{1} f^{\prime}} \right) = 0,$$

17 $$\phi^{\prime\prime} + Scf\phi^{\prime} - ScS_{2} f^{\prime} + \frac{Nt}{{Nb}}\theta^{\prime\prime} - \sigma Sc\left( {1 + \delta \theta } \right)^{n} \exp \left( { - \frac{E}{{\left( {1 + \theta \delta } \right)}}} \right)\phi = 0,$$

18 $$\chi^{\prime\prime} + Lbf\chi^{\prime} - LbS_{3} f^{\prime} - Pe\left( {\left( {\chi + \delta_{1} } \right)\phi^{\prime\prime} + \chi^{\prime}\phi^{\prime}} \right) = 0,$$

19 $$\left\{ {\begin{array}{*{20}l} {f\left( 0 \right) = 0,\,\,\,\,f^{\prime}\left( 0 \right) = 1,\,\,\,\,f^{\prime}\left( \infty \right) \to 0,} \hfill \\ {\theta \left( 0 \right) = 1 - S_{1} ,\,\,\,\,\theta \left( \infty \right) \to 0,} \hfill \\ {\phi \left( 0 \right) = 1 - S_{2} ,\,\,\,\,\phi \left( \infty \right) \to 0,} \hfill \\ {\chi \left( 0 \right) = 1 - S_{3} ,\,\,\,\,\chi \left( \infty \right) \to 0,} \hfill \\ \end{array} } \right\}$$

The emerging parameters are described as:

20 $$\left\{ {\begin{array}{*{20}l} {Rb = \frac{{M_{p} \left( {\rho_{p} - \rho_{f} } \right)\left( {C_{w} - C_{\infty } } \right)}}{{\rho_{p} \rho_{f} \beta^{*} \left( {T_{w} - T_{\infty } } \right)}},\,\,\,\,Rc = \frac{{\gamma \left( {\rho_{m} - \rho_{f} } \right)\left( {N_{w} - N_{\infty } } \right)}}{{\rho_{f} \beta^{*} \left( {T_{w} - T_{\infty } } \right)}},\,\,\,\sigma = \frac{{Kr^{2} }}{a},} \hfill \\ {Rd = \frac{{16\sigma^{*} T_{\infty }^{3} }}{{3k^{*} k_{f} }},\,\,\,\,\Pr = \frac{{\alpha^{*} }}{{\nu_{f} }},\,\,\,\,S_{1} = \frac{{d_{1} }}{{e_{1} }},\,\,\,\,S_{3} = \frac{{d_{3} }}{{e_{3} }},\,\,\,\,Nt = \frac{{\left( {\rho C_{p} } \right)_{p} D_{T} \left( {T_{w} - T_{\infty } } \right)}}{{\left( {\rho C_{p} } \right)_{f} \nu_{f} T_{\infty } }},} \hfill \\ {Nb = \frac{{\left( {\rho C_{p} } \right)_{p} D_{B} M_{p} \left( {C_{w} - C_{\infty } } \right)}}{{\left( {\rho C_{p} } \right)_{f} \nu_{f} }},\,\,\,\,Lb = \frac{{\nu_{f} }}{{D_{m} }},\,\,\,\,E = \frac{{E_{a} }}{{k_{B} T_{\infty } }},\,\,\,\,\delta = \frac{{T_{\infty } }}{{T_{w} - T_{\infty } }},\,\,\,\,} \hfill \\ {M = \frac{{\sigma_{f} B_{0}^{2} }}{{\rho_{f} a}},\,\,\,\,\delta_{1} = \frac{{N_{\infty } }}{{N_{w} - N_{\infty } }},\,\,\,\omega = \frac{{g^{*} \beta^{*} \left( {T_{w} - T_{\infty } } \right)}}{{au_{w} \left( x \right)}},\,\,\,\,Sc = \frac{{\nu_{f} }}{{D_{B} }},\,\,\,\,S_{2} = \frac{{d_{2} }}{{e_{2} }}.} \hfill \\ \end{array} } \right\}$$

where $Rc$ is bio-convective Rayleigh number, $Rb$ is buoyancy ratio factor, $\omega$ is mixed convection factor, $Sc$ and $\Pr$ are Schmidt and Prandtl numbers, $Rd$ is thermal radiation factor, $S_{1}$ is thermal stratification parameter, $S_{2}$ is the concentration stratification factor, $S_{3}$ is the microorganisms stratification factor, $Nt$ is thermophoresis parameter, $Lb$ is bio-convective Lewis number, $Nb$ is Brownian motion factor, $E$ is activation energy parameter, $\delta$ is temperature difference factor, $\delta_{1}$ is microorganisms difference parameter, $M$ is magnetic parameter and $\sigma$ is chemical reaction factor. The default values and ranges of these factors are included in Table 1.

**Table 1:** Embedded parameters.

Parameter	Default value	Range
$\beta$	0.2	0.2–4.0
$Rb$	0.2	0.2–0.4
$Rc$	0.2	0.2–0.4
$\sigma$	1.0	1.0–4.0
$Rd$	0.3	0.3–4.0
$\Pr$	1.0	1.0–4.0
$S_{1}$	0.5	0.2–0.6
$S_{2}$	0.1	0.2–0.6
$S_{3}$	0.1	0.2–0.6
$Nt$	0.5	0.5–4.0
$Nb$	0.5	0.5–4.0
$Lb$	0.5	0.5–4.0
$E$	1.0	1.0–4.0
$\delta$	0.5	0.5
$M$	1.0	0.2–4.0
$\delta_{1}$	0.5	0.5
$Sc$	0.1	0.1–0.4
$\omega$	0.1	0.1–0.3
$Pe$	0.5	0.5–4.0

To discover the surface drag, heat and mass transmission characteristics, and density number, the local quantities of interest expressed as:

21 $$\sqrt {{\text{Re}}_{x} } C_{fx} = \left( {1 + \frac{1}{\beta }} \right)f^{\prime\prime}\left( 0 \right),\,\,\,\,\frac{{Nu_{x} }}{{\sqrt {{\text{Re}}_{x} } }} = - \left( {1 + Rd} \right)\theta^{\prime}\left( 0 \right),\,\,\,\,\frac{{Sh_{x} }}{{\sqrt {{\text{Re}}_{x} } }} = - \phi^{\prime}\left( 0 \right),\,\,\,\,\frac{{Nn_{x} }}{{\sqrt {{\text{Re}}_{x} } }} = - \chi^{\prime}\left( 0 \right),$$

Above ${\text{Re}}_{x} = \frac{{ax^{2} }}{\nu }$ depicts local Reynolds number.

## HAM solution

In this segment, the homotopic solution of the present model is tackled with HAM which is applicable to both linear and nonlinear differential equations. The operators (linear) are given by:

22 $$L_{f} \left( \xi \right) = f^{\prime\prime\prime} - f^{\prime},\,\,\,\,L_{\theta } \left( \xi \right) = \theta^{\prime\prime} - \theta ,\,\,\,\,L_{\phi } \left( \xi \right) = \phi^{\prime\prime} - \phi ,\,\,\,\,L_{\chi } \left( \xi \right) = \chi^{\prime\prime} - \chi .$$

The initial guess for above problem is given by:

23 $$f_{0} \left( \xi \right) = \left( {1 - e^{ - \xi } } \right),\,\,\,\chi_{0} \left( \xi \right) = e^{ - \xi } \left( {1 - S_{3} } \right),\,\,\,\phi_{0} \left( \xi \right) = e^{ - \xi } \left( {1 - S_{2} } \right),\,\,\,\theta_{0} \left( \xi \right) = e^{ - \xi } \left( {1 - S_{1} } \right).$$

With properties:

24 $$L_{f} \left[ {\zeta_{1} + \zeta_{3} e^{\xi } + \zeta_{2} e^{ - \xi } } \right] = 0,\,\,\,\,L_{\theta } \left[ {\zeta_{5} e^{\xi } + \zeta_{4} e^{ - \xi } } \right] = 0,\,\,\,\,L_{\phi } \left[ {\zeta_{7} e^{\xi } + \zeta_{6} e^{ - \xi } } \right] = 0,\,\,\,\,L_{\theta } \left[ {\zeta_{8} e^{ - \xi } + \zeta_{9} e^{\xi } } \right] = 0.$$

where $\zeta_{1} - \zeta_{9}$ are fixed values.

Here 0th deformation problem can be written as:

25 $$\Xi \hbar_{f} N_{f} \left[ {f\left( {\xi ;\Xi } \right),\theta \left( {\xi ;\Xi } \right),\phi \left( {\xi ;\Xi } \right),\chi \left( {\xi ;\Xi } \right)} \right] = \left( {1 - \Xi } \right)L_{f} \left[ {f\left( {\xi ;\Xi } \right) - f_{0} \left( \xi \right)} \right],$$

26 $$\Xi N_{\theta } \hbar_{\theta } \left[ {\theta \left( {\xi ;\Xi } \right),f\left( {\xi ;\Xi } \right),\phi \left( {\xi ;\Xi } \right)} \right] = \left( {1 - \Xi } \right)\,L_{\theta \,} \left[ {\theta \,\,\left( {\xi \,\,;\,\,\Xi } \right) - \theta_{0} \,\,\left( {\,\xi \,} \right)} \right],$$

27 $$\Xi N_{\phi } \hbar_{\phi } \left[ {\phi \left( {\xi ;\Xi } \right),f\left( {\xi ;\Xi } \right),\theta \left( {\xi ;\Xi } \right)} \right] = \left( {1 - \Xi } \right)L_{\phi } \left[ {\phi \,\,\left( {\xi \,;\,\,\Xi \,} \right) - \phi_{0} \left( \xi \right)} \right],$$

28 $$\Xi \hbar_{\chi } N_{\chi } \left[ {\chi \left( {\xi ;\Xi } \right),f\left( {\xi ;\Xi } \right),\phi \left( {\xi ;\Xi } \right)} \right] = \left( {1 - \Xi } \right)L_{\chi } \left[ {\chi \left( {\xi ;\Xi } \right) - \chi_{0} \left( \xi \right)} \right],$$

29 $$\left\{ \begin{gathered} f^{\prime}\left( {0;\Xi } \right) = 1,\,\,\,f\left( {0;\Xi } \right) = 0,\,\,\,\,\,\,\,\,f^{\prime}\left( {\infty ;\Xi } \right) \to 0, \hfill \\ \theta \left( {0\,;\,\,\Xi \,} \right)\,\, = 1 - S_{1} ,\,\,\,\,\theta \left( {\infty \,;\,\,\Xi \,} \right)\,\, \to 0, \hfill \\ \phi \left( {0\,\,;\,\,\Xi } \right)\,\, = 1 - S_{2} ,\,\,\,\,\phi \left( {\infty \,;\,\,\Xi \,} \right) \to 0, \hfill \\ \chi \left( {0\,\,;\,\,\Xi } \right)\,\, = 1 - S_{3} ,\,\,\,\,\chi \left( {\infty \,;\,\Xi \,} \right) \to 0, \hfill \\ \end{gathered} \right\}$$

where $\Xi \in \left[ {0\,\,\,1} \right]$ is the inserting factor, $\hbar$ is auxiliary factor with nonzero value. The $N_{f} ,\,\,N_{\theta } ,\,\,N_{\phi }$ and $N_{\chi }$ are nonlinear and expressded as:

30 $$\begin{aligned} & N_{f} \left[ {f\left( {\xi \,;\,\Xi \,} \right),\theta \left( {\xi \,;\,\Xi \,} \right),\phi \left( {\xi \,\,;\,\,\Xi \,\,} \right),\chi \left( {\xi \,\,;\,\,\Xi \,} \right)} \right] = \left( {1 + \frac{1}{\beta }} \right)\frac{{\partial^{3} f\left( {\xi ;\Xi } \right)}}{{\partial \xi^{3} }} + \frac{{\partial^{2} f\left( {\xi ;\Xi } \right)}}{{\partial \xi^{2} }} \\ & \quad \times f\left( {\xi ;\Xi } \right) - \left( {\,\frac{{\partial f\left( {\xi \,;\,\,\Xi } \right)}}{\partial \xi }\,} \right)^{2} - M\frac{{\partial f\left( {\xi ;\Xi } \right)}}{\partial \xi } + \omega \left( {\theta \left( {\xi ;\Xi } \right) - Rb\phi \left( {\xi ;\Xi } \right) - Rc\chi \left( {\xi ;\Xi } \right)} \right), \\ \end{aligned}$$

31 $$\begin{aligned} & N_{\theta } \left[ {\theta \left( {\xi ;\Xi } \right),f\left( {\xi ;\Xi } \right),\phi \left( {\xi ;\Xi } \right)} \right] = \left( {1 + Rd} \right)\frac{{\partial^{2} \theta \left( {\xi ;\Xi } \right)}}{{\partial \xi^{2} }} + \Pr f\left( {\xi ;\Xi } \right) \\ & \quad \times \frac{{\partial \theta \left( {\xi ;\Xi } \right)}}{\partial \xi } - \Pr S_{1} \frac{{\partial f\left( {\xi ;\Xi } \right)}}{\partial \xi } + Nb\frac{{\partial \theta \left( {\xi ;\Xi } \right)}}{\partial \xi }\frac{{\partial \phi \left( {\xi ;\Xi } \right)}}{\partial \xi } + Nt\left( {\frac{{\partial \theta \left( {\xi ;\Xi } \right)}}{\partial \xi }} \right)^{2} , \\ \end{aligned}$$

32 $$\begin{aligned} & N_{\phi } \left[ {\phi \left( {\xi ;\Xi } \right),f\left( {\xi ;\Xi } \right),\theta \left( {\xi ;\Xi } \right)} \right] = \frac{{\partial^{2} \phi \left( {\xi ;\Xi } \right)}}{{\partial \xi^{2} }} + Scf\left( {\xi ;\Xi } \right)\frac{{\partial \phi \left( {\xi ;\Xi } \right)}}{\partial \xi } - ScS_{2} \\ & \quad \times \frac{{\partial f\left( {\xi ;\Xi } \right)}}{\partial \xi } + \frac{Nt}{{Nb}}\frac{{\partial^{2} \theta \left( {\xi ;\Xi } \right)}}{{\partial \xi^{2} }} - \sigma Sc\left( {1 + \delta \theta \left( {\xi ;\Xi } \right)} \right)\exp \left( { - \frac{E}{{\left( {1 + \delta \theta \left( {\xi ;\Xi } \right)} \right)}}} \right)\phi \left( {\xi ;\Xi } \right), \\ \end{aligned}$$

33 $$\begin{aligned} & N_{\chi } \left[ {\chi \left( {\xi ;\Xi } \right),f\left( {\xi ;\Xi } \right),\phi \left( {\xi ;\Xi } \right)} \right] = \frac{{\partial^{2} \chi \left( {\xi ;\Xi } \right)}}{{\partial \xi^{2} }} + Lbf\left( {\xi ;\Xi } \right)\frac{{\partial \chi \left( {\xi ;\Xi } \right)}}{\partial \xi } \\ & \quad - LbS_{3} \frac{{\partial f\left( {\xi ;\Xi } \right)}}{\partial \xi } - Pe\left( {\left( {\chi \left( {\xi ;\Xi } \right) + \delta_{1} } \right)\frac{{\partial^{2} \phi \left( {\xi ;\Xi } \right)}}{{\partial \xi^{2} }} + \frac{{\partial \chi \left( {\xi ;\Xi } \right)}}{\partial \xi }\frac{{\partial \phi \left( {\xi ;\Xi } \right)}}{\partial \xi }} \right), \\ \end{aligned}$$

For $\Xi = 0$ and $\Xi = 1$ we have:

34 $$\left\{ {\begin{array}{*{20}l} {f\left( {\,\xi \,\,;\,\,1\,\,} \right) = f\left( {\,\,\xi \,\,} \right),\,\,f\left( {\,\xi \,;\,0\,\,} \right) = f_{0} \left( {\,\,\xi \,\,} \right)} \hfill \\ {\theta \left( {\,\,\xi \,\,;\,\,1\,} \right) = \theta \left( {\,\xi \,} \right),\,\,\theta \left( {\,\,\xi \,;\,0\,} \right) = \theta_{0} \,\left( {\,\xi \,\,} \right)} \hfill \\ {\phi \left( {\xi ;0} \right) = \phi_{0} \left( \xi \right),\,\,\,\,\,\,\,\phi \left( {\xi ;1} \right) = \phi \left( \xi \right)} \hfill \\ {\chi \left( {\xi ;0} \right) = \chi_{0} \left( \xi \right),\,\,\,\chi \left( {\xi ;1} \right) = \chi \left( \xi \right)} \hfill \\ \end{array} } \right\}.$$

Using Taylor series expansion, we obtained:

35 $$f\left( {\xi ;\Xi } \right) = f_{0} \left( \xi \right) + \sum\limits_{m = 1}^{\infty } {\,\,\Xi^{m} \,\,\,f_{m} \left( \xi \right)\,\,\,\,\,\,\,where\,\,\,\,\,\,\,\,} f_{m} \left( \xi \right) = \frac{1}{m!}\left. {\frac{{\partial^{m} \,f\left( {\,\xi \,;\,\,\Xi } \right)}}{{\partial \xi \,^{m} }}} \right|_{\Xi = 0} .$$

36 $$\theta \left( {\xi ;\Xi } \right) = \theta_{0} \left( \xi \right) + \sum\limits_{m = 1}^{\infty } {\,\,\Xi^{m} \,\,\theta_{m} \left( \xi \right)\,\,\,\,\,\,\,\,where\,\,\,\,\,\,\,\,} \theta_{m} \left( \xi \right) = \frac{1}{m!}\left. {\frac{{\partial^{m} \,\,\theta \left( {\,\xi \,;\,\,\Xi } \right)}}{{\partial \,\xi \,^{m} }}} \right|_{\Xi = 0} .$$

37 $$\phi \left( {\xi ;\Xi } \right) = \phi_{0} \left( \xi \right) + \sum\limits_{m = 1}^{\infty } {\,\,\Xi^{m} \,\,\phi_{m} \left( \xi \right)\,\,\,\,\,\,\,\,where\,\,\,\,\,\,\,\,} \phi_{m} \left( \xi \right) = \frac{1}{m!}\left. {\frac{{\partial^{m} \,\,\phi \left( {\xi \,\,;\,\,\Xi } \right)}}{{\partial \,\xi^{m} }}} \right|_{\Xi = 0} .$$

38 $$\chi \left( {\xi ;\Xi } \right) = \chi_{0} \left( \xi \right) + \sum\limits_{m = 1}^{\infty } {\Xi^{m} \,\,\chi_{m} \left( \xi \right)\,\,\,\,\,\,\,\,where\,\,\,\,\,\,\,\,} \chi_{m} \left( \xi \right) = \frac{1}{m!}\left. {\frac{{\partial^{m} \,\,\chi \,\left( {\xi \,\,;\,\,\Xi } \right)}}{{\partial \,\,\xi^{m} }}} \right|_{\Xi = 0} .$$

The mth-order deformation problem can be written as:

39 $$L_{f} \left[ {f_{m} \,\,\left( {\xi \,\,} \right) - \,f_{m - 1} \left( {\,\,\xi \,} \right)\zeta_{m} \,\,} \right] = \hbar_{f} \,\,\,R_{f}^{m} \,\,\left( {\,\,\xi \,} \right),$$

40 $$L_{\theta } \left[ {\theta_{m} \left( {\,\xi \,} \right) - \,\,\theta_{m - 1} \left( {\,\xi \,} \right)\zeta_{m} } \right] = \hbar_{\theta } \,\,R_{\theta }^{m} \,\,\left( {\,\,\xi \,\,} \right),$$

41 $$L_{\phi } \left[ {\phi_{m} \left( {\,\xi \,} \right) - \,\phi_{m - 1} \left( {\,\xi \,} \right)\,\,\zeta_{m} } \right] = \hbar_{\phi } \,R_{\phi }^{m} \,\,\left( {\,\,\xi \,} \right),$$

42 $$L_{\chi } \left[ {\chi_{m} \,\left( {\,\xi \,} \right) - \,\chi_{m - 1} \left( {\,\xi \,} \right)\,\,\zeta_{m} } \right] = \hbar_{\chi } \,R\,_{\chi }^{m} \left( {\,\xi \,\,} \right),$$

43 $$\left\{ {\begin{array}{*{20}l} {f_{m} \left( 0 \right) = 0,\,\,\,\,f^{\prime}_{m} \left( 0 \right) = 0,\,\,\,\,f^{\prime}_{m} \left( \infty \right) = 0,} \hfill \\ {\,\,\,\,\,\,\,\,\,\,\,\theta_{m} \left( 0 \right) = 0,\,\,\,\,\theta_{m} \left( \infty \right) = 0,} \hfill \\ {\,\,\,\,\,\,\,\,\,\,\,\phi_{m} \left( 0 \right) = 0,\,\,\,\,\phi_{m} \left( \infty \right) = 0,} \hfill \\ {\,\,\,\,\,\,\,\,\,\chi_{m} \left( 0 \right) = 0,\,\,\,\,\chi_{m} \left( \infty \right) = 0,} \hfill \\ \end{array} } \right\}$$

44 $$R_{f}^{m} \left( \xi \right) = \left( {1 + \frac{1}{\beta }} \right)f^{\prime\prime\prime}_{m - 1} + \sum\limits_{n = 0}^{m - 1} {f_{m - 1 - n} f^{\prime\prime}_{n} } - \sum\limits_{n = 0}^{m - 1} {f^{\prime}_{n} } f^{\prime}_{m - 1 - n} - Mf^{\prime}_{m - 1} + \omega \left( {\theta_{m - 1} - bR\phi_{m - 1} - \chi_{m - 1} Rc} \right),$$

45 $$R_{\theta }^{m} \,\,\left( {\,\xi \,} \right) = \,\theta^{\prime\prime}_{m - 1} \left( {1 + \,Rd\,} \right)\, + \Pr \,\,\sum\limits_{n = 0}^{m - 1} {\,\,\theta^{\prime}_{m - 1 - n} \,f_{n} } - \Pr S_{1} f^{\prime}_{m - 1} + Nb\,\sum\limits_{n = 0}^{m - 1} {\,\,\theta^{\prime}_{m - 1 - n} \,\,\phi^{\prime}_{n} } + N\,t\,\sum\limits_{n = 0}^{m - 1} {\,\,\,\theta^{\prime}_{n} \,\,\theta^{\prime}_{m - 1 - n} } ,$$

46 $$R_{\phi }^{m} \,\,\left( {\,\xi \,} \right) = \phi^{\prime\prime}_{m - 1} + S\,c\,\,\,\sum\limits_{n = 0}^{m - 1} {\phi^{\prime}_{m - 1 - n} \,\,f_{n} } - Sc\,\,\,S_{2} f^{\prime}_{m - 1} + \frac{Nt}{{Nb}}\,\,\theta^{\prime\prime}_{m - 1} - \sigma \,Sc\left( {1 + \delta \,\,\theta_{m - 1} } \right)\exp \,\,\left( { - \frac{E}{{\left( {1\,\,\,\, + \,\,\,\delta \theta_{m - 1} \,} \right)}}\,\,} \right)\phi_{m - 1} ,$$

47 $$R_{\chi }^{m} \,\,\left( {\,\,\xi \,} \right) = \chi^{\prime\prime}_{m - 1} + Lb\,\,\sum\limits_{n = 0}^{m - 1} {\chi^{\prime}_{m - 1 - n} f_{n} } - Lb\,\,S_{3} f^{\prime}_{m - 1} - Pe\left( {\left( {\chi_{m - 1} + \delta_{1} } \right)\,\,\phi^{\prime\prime}_{m - 1} + \sum\limits_{n = 0}^{m - 1} {\phi^{\prime}_{m - 1 - n} \,\,\chi^{\prime}_{n} } } \right),$$

where

48 $$\zeta_{m} = \left\{ {\begin{array}{*{20}l} {0,} \hfill & {when\,\,\,\,m \le 1} \hfill \\ {1,} \hfill & {when\,\,\,\,m > 1} \hfill \\ \end{array} } \right..$$

which is the required solution. The advantages of HAM includes:

- *Convergence Control* HAM allows for control over the convergence of the solution series. The convergence of the solution can be accelerated or improved by adjusting the auxiliary parameter, known as the convergence-control parameter. This flexibility is valuable in obtaining accurate and reliable solutions, especially for highly nonlinear problems.
- *Applicability* HAM is applicable to a wide range of nonlinear differential equations arising in various scientific and engineering fields. It can handle problems with both regular and singular behavior, making it a versatile method for studying diverse phenomena.
- *Efficiency* HAM is computationally efficient compared to some computational approaches. The analytical nature of HAM eliminates the need for discretization of the problem domain, reducing computational efforts and memory requirements.
- *Non-Perturbative Approach* HAM does not rely on perturbation techniques and can capture both weakly and strongly nonlinear behaviors of the system. This makes it a valuable tool for studying problems where traditional perturbation methods may fail.
- *Physical Interpretability* HAM allows for the incorporation of physical parameters and constraints directly into the solution process. This facilitates a deeper understanding of the underlying physical phenomena and provides a physical interpretation of the solution.

## Validation

To validate the results of the current analysis, the HAM results are matched with earlier established results as shown in Table 2. Quite similar results are established here which validate correctness of the current analysis.

**Table 2:** Comparison of current results for $- \theta^{\prime}\left( 0 \right)$ with established results.

$\Pr$	Chen^58^	Zaimi et al.^59^	Sithole et al.^60^	Present results
0.72	0.46315	0.463145	0.46314490	0.463145
1.0	0.58199	0.581977	0.58197671	0.581977
3.0	1.16523	1.165246	1.16524595	1.165246
7.0	1.89537	1.895403	1.89540326	1.895403
10.0	2.30796	2.308004	2.30800394	2.308004

## Discussion of results

**Figure 2:**
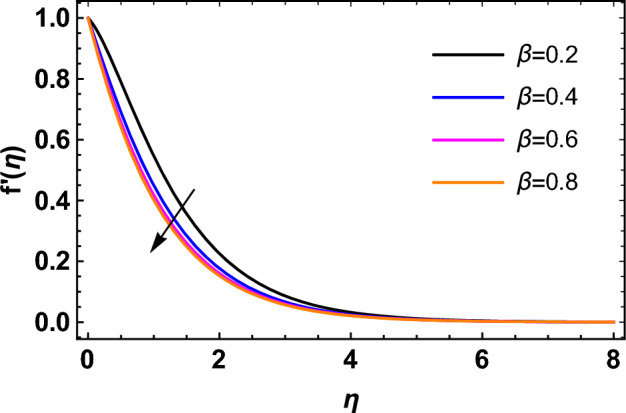
Impression of $\beta$ on $f^{\prime}\left( \eta \right)$.

**Figure 3:**
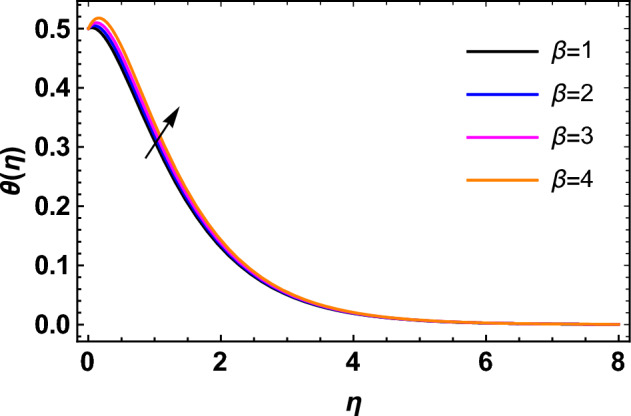
Impression of $\beta$ on $\theta \left( \eta \right)$.

**Figure 4:**
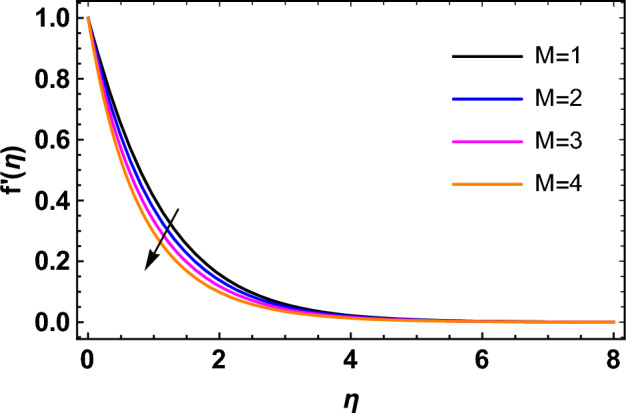
Impression of $M$ on $f^{\prime}\left( \eta \right)$.

**Figure 5:**
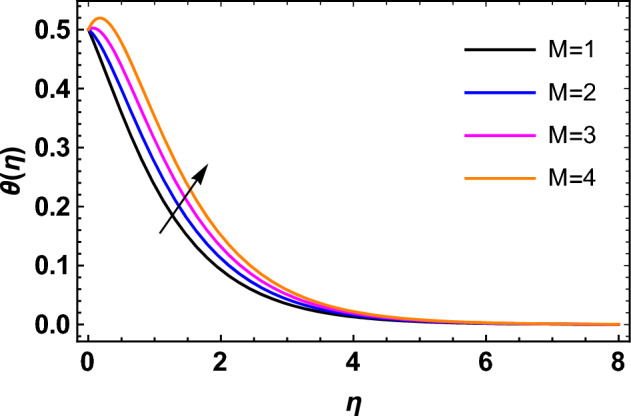
Impression of $M$ on $\theta \left( \eta \right)$.

**Figure 6:**
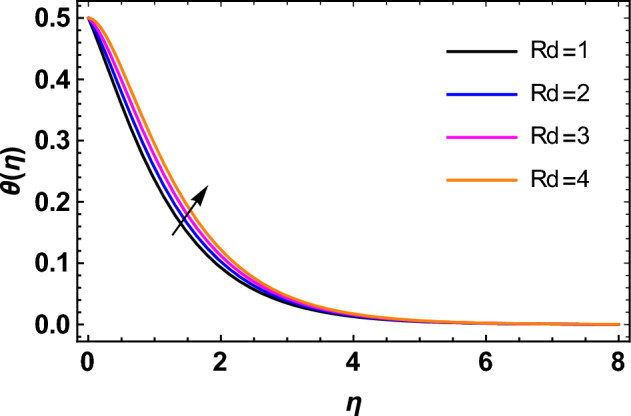
Impression of $Rd$ on $\theta \left( \eta \right)$.

**Figure 7:**
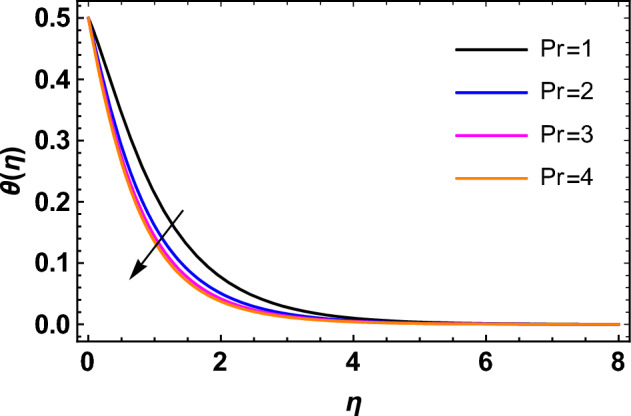
Impression of $\Pr$ on $\theta \left( \eta \right)$.

**Figure 8:**
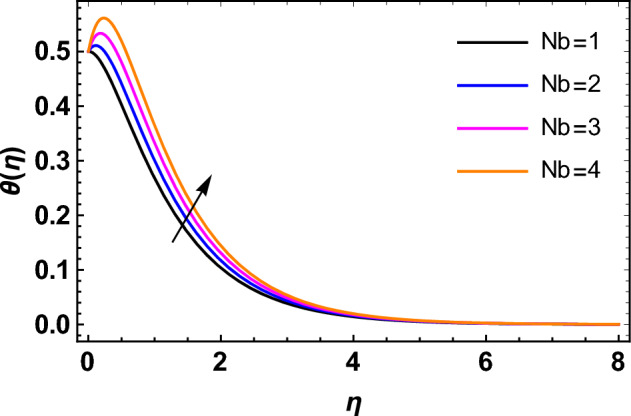
Impression of $Nb$ on $\theta \left( \eta \right)$.

**Figure 9:**
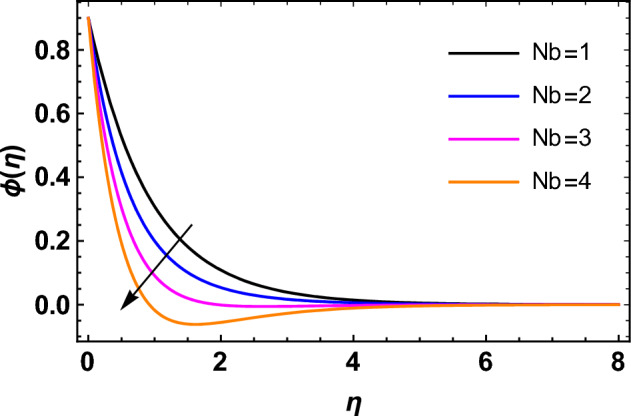
Impression of $Nb$ on $\phi \left( \eta \right)$.

**Figure 10:**
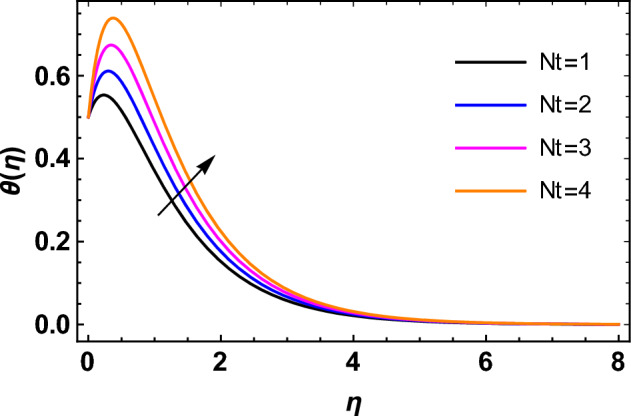
Impression of $Nt$ on $\theta \left( \eta \right)$.

**Figure 11:**
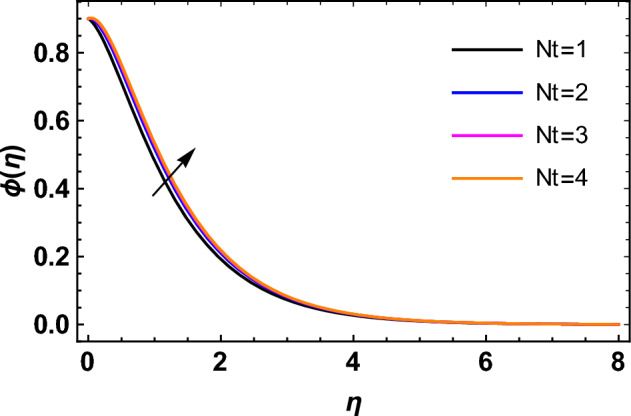
Impression of $Nt$ on $\phi \left( \eta \right)$.

**Figure 12:**
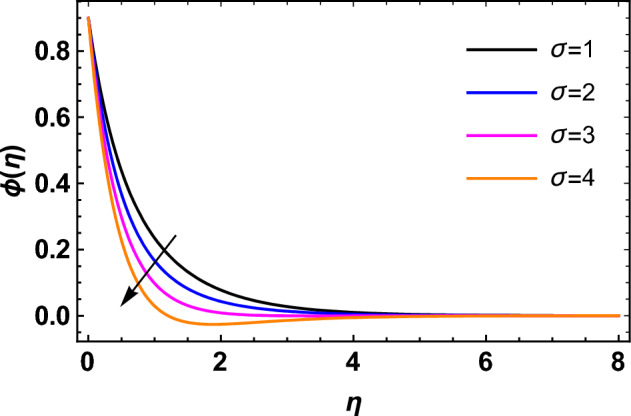
Impression of $\sigma$ on $\phi \left( \eta \right)$.

**Figure 13:**
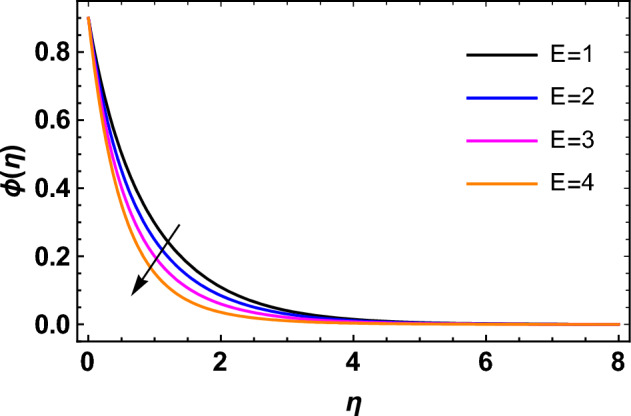
Impression of $E$ on $\phi \left( \eta \right)$.

**Figure 14:**
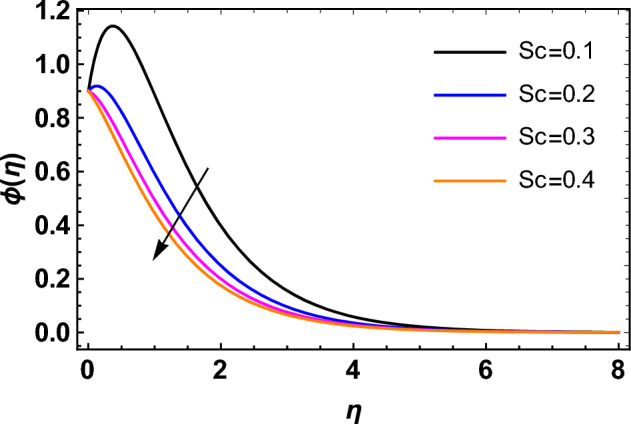
Impression of $Sc$ on $\phi \left( \eta \right)$.

**Figure 15:**
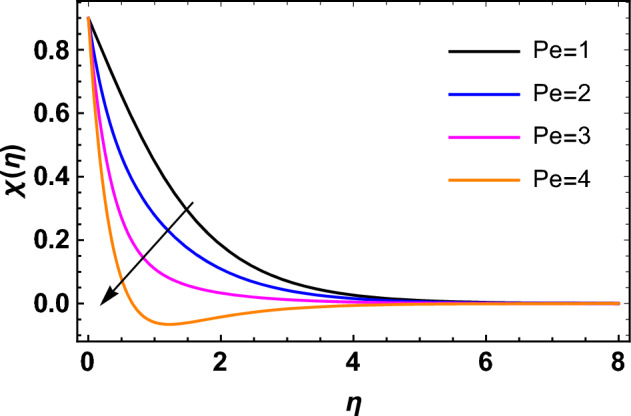
Impression of $Pe$ on $\chi \left( \eta \right)$.

**Figure 16:**
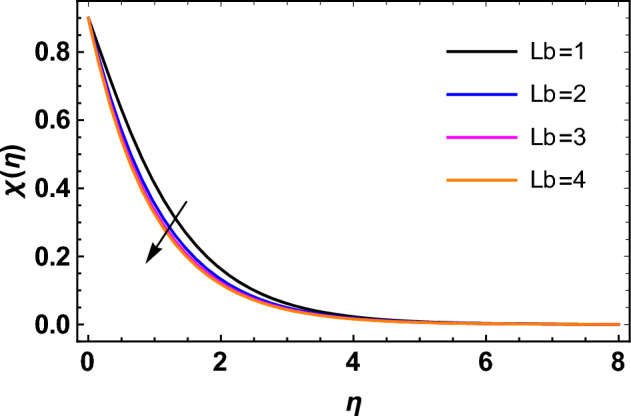
Impression of $Lb$ on $\chi \left( \eta \right)$.

This segment deals with the influences of emerging factors on various flow profiles using numerous figures. Additionally, the impressions of emerging factors on the skin friction, Sherwood, Nusselt, and density numbers are exhibited by means of Tables. The default values of the embedded factors are shown in Table 1. Figures 2, 3 demonstrate the consequence of Casson factor ($$\beta$$) on velocity ($$f^{\prime}\left( \eta \right)$$) and temperature ($$\theta \left( \eta \right)$$). The increasing $$\beta$$ reduces both $$f^{\prime}\left( \eta \right)$$ and $$\theta \left( \eta \right)$$. It is well known that the increasing $$\beta$$ expands the fluid’s viscosity which causes reduction in velocity of fluid. Therefore, the growth in $$\beta$$ diminishes the fluid velocity. Also, the increasing $$\beta$$ reduces the yield stress which consequently weakens the thickness of thermal boundary layer. Therefore, the increasing $$\beta$$ reduces thermal profile of Casson fluid flow as displayed in Fig. 3. Figures 4, 5 display the consequences of $$M$$ on $$f^{\prime}\left( \eta \right)$$ and $$\theta \left( \eta \right)$$, respectively. The growing $$M$$ reduces $$f^{\prime}\left( \eta \right)$$ while increases $$\theta \left( \eta \right)$$. The greater $$M$$ increases the dragging force on extending sheet surface and weakens velocity panels. This effect occurs due the Lorentz force that encounters the fluid particles flow. Thus, the increasing magnetic parameter reduces the Casson fluid flow whereas the increasing dragging force on elongating sheet surface upsurges the thickness of thermal layer at the boundary. The intensification in the boundary of thermal layer results augmentation in the temperature fluid. Therefore, the growing magnetic factor augments the thermal panel. Figure 6 demonstrates the impact of radiative factor ($$Rd$$) on $$\theta \left( \eta \right)$$. The increasing thermal radiation factor significantly augments $$\theta \left( \eta \right)$$. It is obvious that the aggregating $$Rd$$ upsurge the thermal panels. This outcome is due to the reason that as $$Rd$$ increase then the Rosseland radiative absorptivity ($$k^{*}$$) (from the definition of $$Rd$$) which results an augmentation in rate of thermal flow and escalates the temperature of the Casson fluid flow. Hence, aggregating thermal radiation factor increases $$\theta \left( \eta \right)$$. Figure 7 depicts the impression of Prandtl number ($$\Pr$$) on $$\theta \left( \eta \right)$$. Thermal distribution along with thermal layer at the boundary monotonically declines as $$\Pr$$ rises. The reason behind this effect is because when $$\Pr$$ rises, the thermal boundary layer thickness reduces as growth in $$\Pr$$ corresponds to a thinner boundary layer and a weaker thermal diffusivity. Figures 8, 9 show the influence of Brownian motion factor ($$Nb$$) upon $$\theta \left( \eta \right)$$ and $$\phi \left( \eta \right)$$, respectively. The thermal characteristics augment while the concentration distribution upsurges with hike in $$Nb$$. Here, we can see that $$\theta \left( \eta \right)$$ grows as the Brownian parameter factor $$Nb$$ increases. This phenomenon showed that Brownian motion, which produces micro-mixing and increases a nanofluid's thermal conductivity, is primarily responsible for the increasing Casson fluid flow temperature (see Fig. 8). Conversely, the greater $$Nb$$ reduces the concentration profile significantly. The reason is that when particle Brownian motion increases, the fluid moves irregularly and is vigorously mixed that causes in degeneration in the nanoparticle concentration distribution of Casson fluid as displayed in Fig. 9. Figures 10 and 11 depict the impression of thermophoresis factor ($$Nt$$) over $$\theta \left( \eta \right)$$ and $$\phi \left( \eta \right)$$, respectively. Both these profiles augment with the increasing thermophoresis factor ($$Nt$$). Increased $$Nt$$ values result in an enrichment of the thermophoresis force, which in turn leads nanoparticles to diffuse into the surrounding fluid owing to temperature gradients, thickening thermal and concentration boundary layers. It is obvious that a rise in the thermophoretic effect indicates better penetration of nanoparticles in the surrounding fluid that cause an upsurge in thermal and concentration characteristics of the Casson fluid flow. Furthermore, shear thinning fluids are dominated by temperature and concentration layers features at boundary. Figure 12 indicates the impact of chemical reaction factor ($$\sigma$$) on $$\phi \left( \eta \right)$$. The higher values of ($$\sigma$$) reduces $$\phi \left( \eta \right)$$. Physically, this stands to the reason as the destructive chemical reaction speeds up the rate at which reactant species decompose and reduces $$\phi 
\left( \eta \right)$$. Figure 13 displays the influence of activation energy factor ($$E$$) on concentration profile. The upsurge in ($$E$$) augments the concentration panels. Increasing $$E$$ retards the Arrhenius function and boosts the chemical reaction effects that generates the boundary layer's high concentration. So, the increasing $$E$$ augments $$\phi \left( \eta \right)$$ of the Casson fluid. Figure 14 exhibits the impact of $$Sc$$ on $$\phi \left( \eta \right)$$. An upsurge in $$Sc$$ retards $$\phi \left( \eta \right)$$. Greater values of $$Sc$$ indicate that the fluid has a lower chemical molecular diffusivity, or that mass transport contributes less to diffusion. Therefore, with growth in $$Sc$$, the thickness of the concentration boundary layer thickness decreases. Greater species diffusion takes place with lower values of $$Sc$$, and the thickness of concentration layer at the boundary rises. It follows that under such an environment, a lower Schmidt number diffusing species must be used to improve concentration profile in the medium, according to chemical engineering designers. Figure 15 indicates the influence of Peclet number ($$Pe$$) on microorganisms profile. The growing Peclet number reduces the microorganisms profile. There is an inverse relation amongst the Peclet number and microorganism diffusivity, and there is a direct relation between the Peclet number and cell swimming speed and chemotaxis constant. The Peclet number is associated with microorganisms diffusivity, means that the greater Peclet number reduces the microorganisms diffusivity and as a result the density profile reduces. Therefore, the microorganisms profile of the Casson fluid flow diminishes for higher Peclet number. Figure 16 indicates the impact of $$Lb$$ on microorganisms profile ($$\chi \left( \eta \right)$$). The increasing value of $$Lb$$ reduces the microorganisms profile. Table 3 shows the influence $$\omega$$, $$Rb$$, $$Rc$$ and $$M$$ on surface drag of the Casson fluid flow. Form Table 3, it is found that the increasing $$\omega$$ and $$Rc$$ reduces the surface drag of Casson fluid flow. Conversely, the aggregating values of $$Rb$$ and $$M$$ augments the surface drag of the Casson fluid flow. Table 4 shows the influence $$Nt$$, $$Nb$$, $$Rd$$ and $$S_{1}$$ on the rate of heat transfer of the fluid. From Table 4, it is found that the boosting values of $$Nt$$ and $$Nb$$ diminishes the rate of thermal flow of fluid whereas the increasing $$Rd$$ and $$S_{1}$$ increases rate of thermal flow of fluid. Table 5 shows the consequences of $$Nt$$, $$Nb$$, $$Sc$$ and $$S_{2}$$ on rate of mass transfer. From here, it is noticed that increasing $$Nt$$ reduces the mass transfer rate of the Casson fluid flow while the increasing $$Nb$$, $$Sc$$ and $$S_{2}$$ augments the mass transfer rate for fluid. Table 6 shows the impact of $$Lb$$, $$Pe$$ and $$S_{3}$$ on density number of the Casson fluid flow. From this Table, it is found that the augmenting $$Lb$$ and $$S_{3}$$ increases the density number of the Casson fluid while the upsurge in $$Pe$$ diminishes the density number of the Casson fluid flow.

**Table 3:** Influences of the physical parameters on $\left( {1 + \frac{1}{\beta }} \right)f^{\prime\prime}\left( 0 \right)$.

$\omega$	$Rb$	$Rc$	$M$	$\left( {1 + \frac{1}{\beta }} \right)f^{\prime\prime}\left( 0 \right)$
0.1				− 0.9956
0.2				− 0.9600
0.3				− 0.9240
	0.2			− 0.9445
	0.3			− 0.9650
	0.4			− 0.9856
		0.2		− 0.9183
		0.3		− 0.9127
		0.4		− 0.9071
			0.2	− 0.9816
			0.6	− 1.2022
			0.9	− 1.3579

**Table 4:** Influences of the physical parameters on $- \left( {1 + Rd} \right)\theta^{\prime}\left( 0 \right)$.

$Nt$	$Nb$	$Rd$	$S_{1}$	$- \left( {1 + Rd} \right)\theta^{\prime}\left( 0 \right)$
0.5				0.4570
0.7				0.4049
0.9				0.3694
	0.5			0.6131
	0.7			0.5651
	0.9			0.5161
		0.3		0.7474
		0.6		0.8258
		0.9		0.8921
			0.2	0.9050
			0.4	0.9109
			0.6	0.9225

**Table 5:** Influences of the physical parameters on $- \phi^{\prime}\left( 0 \right)$.

$Nt$	$Nb$	$Sc$	$S_{2}$	$- \phi^{\prime}\left( 0 \right)$
0.5				0.1988
0.7				0.0867
0.9				0.0089
	0.5			1.0969
	0.7			1.1234
	0.9			1.1412
		0.1		0.0536
		0.3		0.1634
		0.5		0.2720
			0.2	0.7226
			0.4	0.9755
			0.6	1.2201

**Table 6:** Influences of the physical parameters on $- \chi^{\prime}\left( 0 \right)$.

$Lb$	$Pe$	$S_{3}$	$- \chi^{\prime}\left( 0 \right)$
0.5			0.4570
0.6			0.5178
0.7			0.5753
	0.5		0.3537
	0.6		0.3438
	0.7		0.3325
		0.2	0.3398
		0.4	0.3467
		0.6	0.4159

## Conclusion

In this section, the final outcomes of the laminar, steady, and incompressible MHD flow of a non-Newtonian Casson fluid flow over a stratified stretching sheet are presented. A semi-analytical investigation along with validation with previous results is presented. The final outcomes are listed as:

- When the Casson and magnetic factors increase, the velocity distribution of the Casson fluid flow diminishes.
- As the Casson factor and Prandtl number rise, temperature distribution of fluid flow diminishes. On the other hand, when thermal radiation, magnetic, and Brownian motion factors rise, the temperature distribution of the fluid also rises.
- The concentration distribution of fluid is reduced by the higher Schmidt number, Brownian motion, and chemical reaction factor whereas the increased thermophoresis and activation energy parameters enhance the Casson fluid flow concentration.
- The microorganism profile is reduced when the bioconvection Peclet and Lewis numbers rise.
- It is perceived that when the bioconvective Rayleigh number and mixed convection factor increase, the surface drag of the Casson fluid flow decreases while, the rising buoyancy ratio factor and the magnetic factor increase the surface drag.
- The rate of heat transmission is decreased by Brownian motion and rising thermophoresis whereas the rate of heat transfer is accelerated by rising thermal radiation and thermal stratification factors.
- It is found that raising the thermophoresis factor lowers the mass transmission rate while increasing the Brownian factor, Schmidt number, and concentration stratification factor raises the heat transfer rate.

## Data Availability

The data that support the findings of this study are available from the corresponding author upon reasonable request.

## List of symbols

$$x,y$$: Coordinates
$$a$$, $$e_{1}$$, $$e_{2}$$, $$e_{3}$$, $$d_{1}$$, $$d_{2}$$, $$d_{3}$$: Constants
$B_{0}$: Strength of magnetic field
$\mu_{{{\text{Bnf}}}}$: Plastic dynamic viscosity
$$P_{y}$$: Yield stress
$$\nabla$$: Differential operator
$$\nu_{f}$$: Kinematic viscosity
$\rho_{f}$: Density of the nanofluid
$$\sigma_{f}$$: Electrical conductivity
$$\beta$$: Casson parameter
$$g^{*}$$: Gravitational acceleration
$\beta^{*}$: Thermal expansion coefficient
$M_{p}$: Molar mass of nanoparticle
$$\rho_{p}$$: Nanoparticle density
$\gamma^{*}$: Volume of the microorganisms
$$N$$: Microorganisms concentration
$$C$$: Nanoparticles concentration
$$T$$: Temperature
$$k_{f}$$: Thermal conductivity
$$C_{p}$$: Specific heat
$\sigma^{*}$: Stefan–Boltzmann constant
$k^{*}$: Mean absorption coefficient
$Q_{0}$: Heat source coefficient
$$D_{B}$$: Brownian diffusivity
$$D_{T}$$: Thermophoretic coefficient
$K_{r}$: Coefficient of chemical reaction
$$n$$: Power index
$Rc$: Bioconvection Rayleigh number
$\omega$: Mixed convection factor
$Rb$: Buoyancy ratio factor
$Rd$: Thermal radiation factor
$$Sc$$: Schmidt number
$E$: Activation energy parameter
$$\Pr$$: Prandtl number
$S_{1}$: Thermal stratification factor
$S_{2}$: Concentration stratification factor
$S_{3}$: Microorganisms stratification factor
$Nb$: Brownian motion factor
$$M$$: Magnetic parameter
$Lb$: Bioconvection Lewis number
$\delta$: Temperature difference factor
$\delta_{1}$: Microorganisms difference parameter
$\sigma$: Chemical reaction parameter
